# System interoperability and data linkage in the era of health information management: A bibliometric analysis

**DOI:** 10.1177/18333583241277952

**Published:** 2024-09-16

**Authors:** Tiago Costa, Teresa Borges-Tiago, Francisco Martins, Flávio Tiago

**Affiliations:** 1 School of Business and Economics, University of the Azores, Ponta Delgada, Azores, Portugal; 2 Pharmaceutical Services, Unidade de Saúde da Ilha de São Miguel, Ponta Delgada, Azores, Portugal; 3 Faculty of Science and Technology, University of the Azores, Ponta Delgada, Azores, Portugal; 4 Centre of Applied Economics Studies of the Atlantic (CEEAplA), Ponta Delgada, Azores, Portugal

**Keywords:** health information management, health information interoperability, health information systems, medical informatics, health information exchange, information storage and retrieval, bibliometrics, health information governance, data linkage, healthcare stakeholders, system interoperability, e-health, blockchain in healthcare

## Abstract

**Background:** Across the world, health data generation is growing exponentially. The continuous rise of new and diversified technology to obtain and handle health data places health information management and governance under pressure. Lack of data linkage and interoperability between systems undermines best efforts to optimise integrated health information technology solutions. **Objective**: This research aimed to provide a bibliometric overview of the role of interoperability and linkage in health data management and governance. **Method**: Data were acquired by entering selected search queries into Google Scholar, PubMed, and Web of Science databases and bibliometric data obtained were then imported to Endnote and checked for duplicates. The refined data were exported to Excel, where several levels of filtration were applied to obtain the final sample. These sample data were analysed using Microsoft Excel (Microsoft Corporation, Washington, USA), WORDSTAT (Provalis Research, Montreal, Canada) and VOSviewer software (Leiden University, Leiden, Netherlands). **Results**: The literature sample was retrieved from 3799 unique results and consisted of 63 articles, present in 45 different publications, both evaluated by two specific in-house global impact rankings. Through VOSviewer, three main clusters were identified: (i) e-health information stakeholder needs; (ii) e-health information quality assessment; and (iii) e-health information technological governance trends. A residual correlation between interoperability and linkage studies in the sample was also found. **Conclusion**: Assessing stakeholders’ needs is crucial for establishing an efficient and effective health information system. Further and diversified research is needed to assess the integrated placement of interoperability and linkage in health information management and governance. **Implications**: This research has provided valuable managerial and theoretical contributions to optimise system interoperability and data linkage within health information research and information technology solutions.

## Introduction

The continuous generation of enormous amounts of health data poses several challenges to its management and governance. Currently, healthcare stakeholders have access to raw health data that must be processed to enable value creation (Arul et al., 2024; Genevieve et al., 2019). Healthcare systems adopt informatics systems that allow the analysis of health data retrieved and provided by different stakeholders (Genevieve et al., 2019). However, there is a lack of linkage and interoperability between these systems (Bates, 2005; Edwards et al., 2010; Genevieve et al., 2019; van Olmen et al., 2020).

As different and complex health informatics systems emerge, the need for integrating and linking data from various datasets also emerges. Data linkage can be defined as “a process of pairing records from two files and trying to select the pairs that belong to the same entity” (Bohensky et al., 2010; Winglee et al., 2005). The Organisation for Economic Co-operation and Development also defined data linkage as “a merging that brings together from two or more sources of data with the object of consolidating facts concerning an individual or an event that are not available in any separate record” (Harron, 2016). In the healthcare sector, data linkage has been applied, for example, to the integration of patient health records and death certificates (Christen, 2019). Other general health-related applications include epidemiological, managerial and service production studies (Holman et al., 2008). Data linkage’s purpose is to enable the integration of different datasets considering the identification and interconnection of records within an organisation with single datasets or multiple ones (Christen, 2019; Green et al., 2015). The multitude of settings and operations within healthcare information systems can threaten the obtainment of optimised linkage between diversified data libraries. For this reason, stakeholders need to continuously address the problem of interconnectivity between healthcare information systems to find facilitators and solutions (Hopf et al., 2014, 2016; March et al., 2020).

With the need for data linkage also comes the need for interoperability between systems. Interoperability is currently one of the top targets of researchers in the field of information technology (Torab-Miandoab et al., 2023). According to the IEEE Standard Computer Dictionary, interoperability is “the ability of two or more systems or components to exchange information and to use the information that has been exchanged” (Lehne et al., 2019). As different hospitals and physicians are increasing their adoption of digital health data records, the lack of interoperability between healthcare informatics systems may pose difficulties regarding communication processes (Lehne et al., 2019; Reisman, 2017). Therefore, healthcare stakeholders must promote research in this field, trying to understand and identify the core variables within different social, political and clinical challenges and contexts, which can be crucial for establishing an interoperable system. Moreover, there is an awareness of the need for a universal interoperability strategy between all healthcare stakeholders (Tiago et al., 2016). Other aspects, such as technology architecture, system governance and core dataset definition, may be crucial for implementing successfully an interoperable system (Azarm et al., 2017). Interoperability standards are also key in any functioning interoperable system (Gowda et al., 2022). Some benefits of interoperable systems include facilitated access to patient health data, more understanding of medical terms, medical bias minimisation, improved health cost management and integration of diversified types of health data (Iroju et al., 2013). However, barriers to interoperability implementation are still present and include the complexity of the healthcare environment, the lack of standardisation, the existence of legacy systems (e.g. an outdated electronic health record system that does not comply with current standards) and semantic compatibilisation issues (e.g. two systems that cannot recognise and interpret each other’s information) and resistance to change to digitalisation processes (Iroju et al., 2013).

### Current study

System interoperability and data linkage issues span different types of healthcare environments, leading to difficulties in accessing patient health data generated in different settings. Research made about these concerns needs to be verified to evaluate the state-of-the-art of relevant potential needs and trends. The aim of the current research was to provide an overview of the current and future role of data linkage and system interoperability within the domain of health information management and governance by conducting a bibliometric analysis of relevant published literature.

## Method

### Search strategy

This bibliometric analysis was based on a search of research articles across three platforms: Google Scholar, PubMed and Web of Science, considered to be the most relevant databases for this analysis. Search terms used for each platform were: “health data management,” “health information governance,” “health information management,” “healthcare data governance,” “healthcare data management,” “healthcare information governance,” “healthcare information management,” associated with the terms “linkage” and “interoperability” (Supplemental material Table S1, online supplement). No year limitations were applied as the objective was to maximise the analysis of the evolution of publishing research articles over time.

### Data acquisition

Data were collected from the three search platforms between December 2021 and February 2022. Search results from Google Scholar were first added to the “My Library” feature and then retrieved to an EndNote file using the export function. From the Web of Science search platform (Web of Science Core Collection), data were retrieved directly to an EndNote file. For PubMed, data were obtained using its citation manager, which created a compatible EndNote file. All data were extracted during February 2022. For the citation analysis, the number of citations per article was retrieved manually after completion of the search, and on the same day from Google Scholar.

### Data selection process and analysis

**Figure 1.**
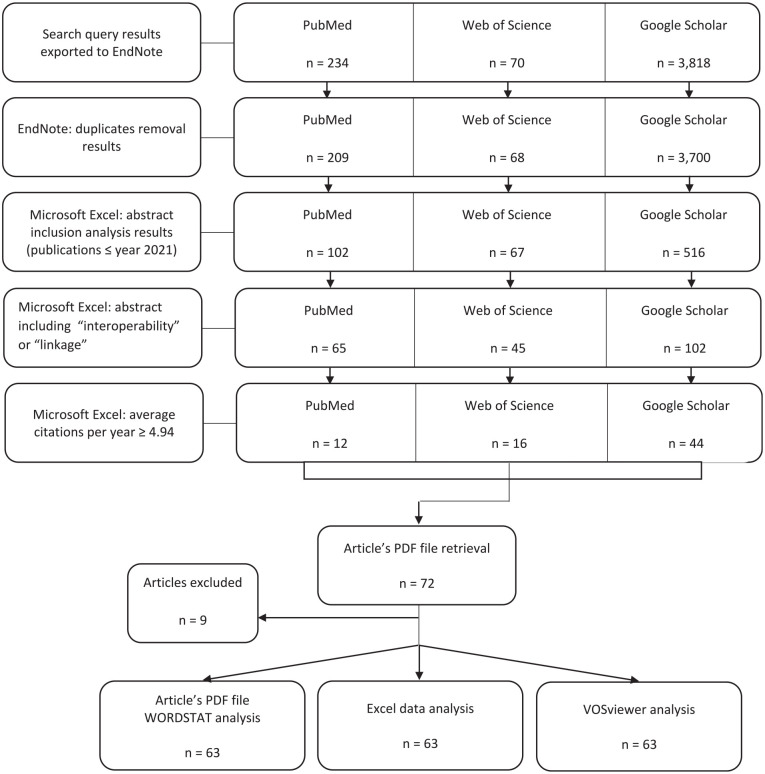
Data selection process and analysis.

All reference files obtained through each search platform were imported to EndNote, followed by a reference update using EndNote’s “Find Reference Updates” feature. With up-to-date references, another automatic EndNote function was used, namely “Find Duplicates,” to eliminate duplicated references. The refined data obtained in EndNote were transferred into Microsoft Excel (Microsoft Corporation, Washington, USA) for further analyses (Figure 1). Using this software, an article’s abstract analysis was performed. The first stage was to verify which results had an abstract and to eliminate the articles without abstracts. At this stage, only articles published by the end of 2021 were considered, as 2022 was an incomplete year and could have provided subsequent erroneous citation calculations and results. In the second stage, selected abstracts were analysed for the inclusion of the term “interoperability” or “linkage.” The results (articles with abstracts that included one or both of these terms) were then analysed according to their average yearly citations. The baseline value of the previous metric was calculated by the average number of citations and article years of the 212 selected articles, to narrow and obtain even more meaningful and impacting results. Research articles with a citation average of less than 4.94 per year were excluded, as this value represented the minimum specific threshold within the scope of the analysis of articles considered impactful (Emmer et al., 2022). Finally, the selected 72 references were screened to verify they met the criteria for the scope of analysis (through screening of articles’ PDF files). Following the earlier selection process, data were evaluated using mainly Microsoft Excel, WORDSTAT v. 9.0.11 and VOSviewer v. 1.6.18 software as schematised in Figure 1. VOSviewer tools (Leiden University, Leiden, Netherlands) were deployed to verify potential relationships between research terms and the strength of these relationships within the abstracts and article titles, and to assess their chronological order.

## Results

### Overview of the data acquisition and selection process

**Figure 2.**
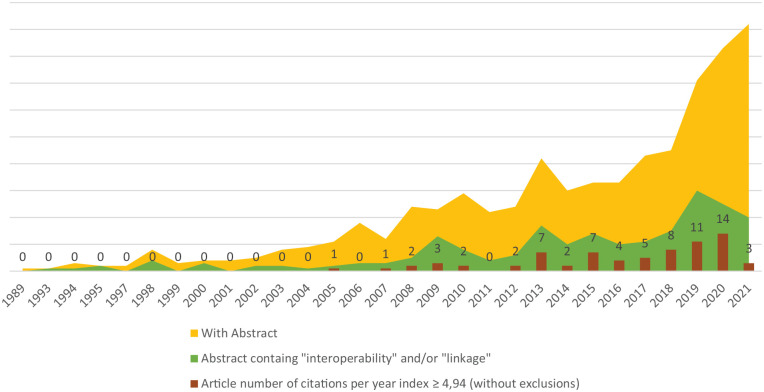
Chronological evolution of publications by level of data selection.

Search results were refined through several processes, which led to the final sample. One of the first meaningful levels of data treatment was the abstract inclusion criterion, which revealed that from the 3977 unique references obtained in EndNote, only 685 (17.22%) had an abstract included in the exported results. After identifying the abstracts that included the terms, “interoperability” and/or “linkage,” the number dropped to 212 references (30.95%; 5.33% from the 3977 unique references). When the last criterion was applied (average citations per year ⩾4.94), only 72 references (33.96%; 1.81% of the 3977 unique references) remained. As to type of publications identified during the sample selection process, most were journal articles (Supplemental material Table S2, online supplement). In terms of chronological evolution (Figure 2), there were few publications on this research topic before 2005. From 2005, the number of publications continued to rise, reaching its peak between 2018 and 2021. In 2019, the number of refined publications (those with an abstract; and those with an abstract that included the selected terms) increased significantly. However, from 2020, these two publication categories began to diverge, as the number of publications with abstracts that included the selected terms continued to drop until the end of 2021.

### Sample overview

**Figure 3.**
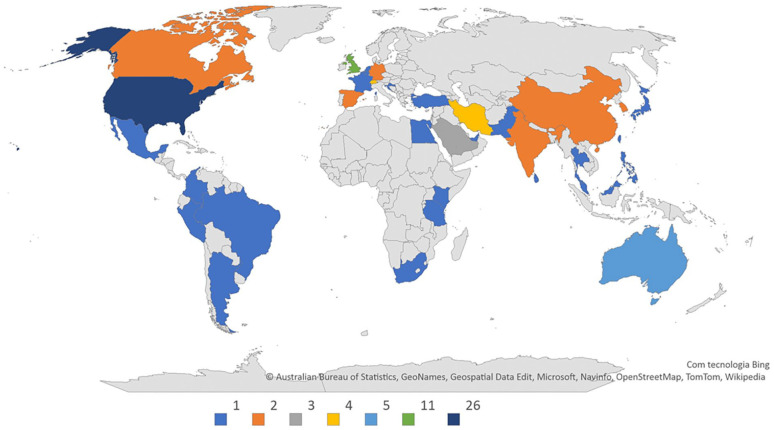
Geographical distribution of articles according to their authors’ origin.

According to our research methodology, three search platforms were chosen to provide results on our topic of analysis. Selected articles were searchable heterogeneously throughout each platform. Most articles were available on Google Scholar (63; 100%) and PubMed (58; 92%), while Web of Science results were scarce (14; 22%). As previously noted, 72 articles were selected for the preliminary sample. A detailed analysis of these research articles led to the exclusion of a further nine articles, which did not fit the scope of the present study. The remaining 63 selected articles are described and summarised in Supplemental material Table S3 (online supplement). Of these 63 articles, 51 (80.95%) contained the word “interoperability” in the abstract, followed by the word “linkage” with 11 (17.46%) results, and only 1 (1.59%) article had an abstract containing both “interoperability” and “linkage.” The average number of authors per article was 5, and the maximum and minimum number of authors per article ranged between 15 and 1, respectively. Most articles had between 2 and 5 authors (48 articles; 76.19%). According to Figure 3, 35 countries were associated with at least 1 article. The country with the highest representation was the United States, with 26 associated articles (41.27%), followed by the United Kingdom with 11 articles (17.46%) and Australia with 5 articles (7.94%). Iran and Switzerland were each represented in four articles, and Saudi Arabia in three articles. Six countries (Canada, China, Germany, India, South Korea and Spain) were each represented in 2 research articles, while 23 countries were each represented in only 1 article.

### Article citation analysis

According to the data retrieved and exported to Table 1, the highest number of citations was achieved by Koppel and Lehmann (2015) and the lowest by Ammar et al. (2021). As for the average number of citations per year, Gordon and Catalini (2018) had the highest average and Ammar et al. (2021) the lowest. The average number of citations of all 63 articles was 101.38, while the mean value of the average number of citations per year was 17.19. A reference global impact ranking was also established, which resulted from the multiplication of the average number of citations by the average number of citations per year. The top five publications of this ranking were: (1st) Gordon and Catalini (2018); (2nd) Mandel et al. (2016); (3rd) Koppel and Lehmann (2015); (4th) Kaplan and Harris-Salamone (2009) and (5th) Detmer et al. (2008). The lowest five included the references Genevieve et al. (2019), Ammar et al. (2020), Samra et al. (2020), Arul et al. (2024) and Ammar et al. (2021).

**Table 1.** Reference characterisation and global impact ranking.

References	Publication type	No. of citations (a)	Average citations per year (b)	Global impact ranking (a) × (b)
Gordon and Catalini (2018)	Journal Article	472	118	1
Mandel et al. (2016)	Journal Article	475	79.17	2
Koppel and Lehmann (2015)	Journal Article	513	38.69	3
Kaplan and Harris-Salamone (2009)	Journal Article	503	38.69	4
Detmer et al. (2008)	Journal Article	482	34.43	5
Ford et al. (2009)	Journal Article	393	30.23	6
Ahmed et al. (2020)	Journal Article	142	71	7
Bahga and Madisetti (2013)	Journal Article	217	24.11	8
Bates (2005)	Journal Article	291	17.11	9
Hussien et al. (2019)	Journal Article	103	34.33	10
Dinh-Le et al. (2019)	Journal Article	99	33	11
Jones et al. (2014)	Journal Article	161	20.23	12
Huang et al. (2017)	Journal Article	104	20.8	13
Furukawa et al. (2013)	Journal Article	136	15.11	14
Sittig et al. (2018)	Journal Article	79	19.75	15
Hylock and Zeng (2019)	Journal Article	68	22.67	16
Johnson et al. (2008)	Journal Article	117	12.64	17
Lavin et al. (2015)	Journal Article	94	13.43	18
Kalkman et al. (2019)	Journal Article	54	18	19
Mohammadzadeh and Safdari (2014)	Journal Article	88	11	20
Rezaeibagha et al. (2015)	Journal Article	82	11.71	21
Dubovitskaya et al. (2020)	Journal Article	43	21.5	22
Witry et al. (2010)	Journal Article	105	8.75	23
Krittanawong et al. (2020)	Journal Article	41	20.5	24
Jones et al. (2019)	Journal Article	50	16.67	25
Lucyk et al. (2017)	Journal Article	64	12.8	26
Vazirani et al. (2020)	Journal Article	39	19	27
Alonso et al. (2019)	Journal Article	45	15	28
Salas-Vega et al. (2015)	Journal Article	68	9.71	29
Blasimme et al. (2018)	Journal Article	50	12.5	30
Lee et al. (2020)	Journal Article	35	17.5	31
Warner et al. (2016)	Journal Article	59	9.83	32
Kharrazi et al. (2017)	Journal Article	53	10.6	33
Downs et al. (2019)	Journal Article	38	12.67	34
Blazona and Koncar (2007)	Journal Article	83	5.53	35
Kasthurirathne et al. (2015)	Journal Article	56	8	36
Edwards et al. (2010)	Journal Article	71	5.92	37
Masud et al. (2012)	Journal Article	63	6.3	38
Vest (2012)	Journal Article	60	6	39
Avila et al. (2017)	Journal Article	42	8.4	40
Oderkirk et al. (2013)	Journal Article	56	6.22	41
Durneva et al. (2020)	Journal Article	26	13	42
Sethi and Laurie (2013)	Journal Article	55	6.11	43
Wollersheim et al. (2009)	Journal Article	66	5.08	44
Ahmadi and Aslani (2018)	Journal Article	36	9	45
Abdekhoda et al. (2016)	Journal Article	42	7	46
Sinaci et al. (2020)	Journal Article	24	12	47
Alkraiji et al. (2013)	Journal Article	49	5.44	48 = 49
Tapuria et al. (2013)	Journal Article	49	5.44	49 = 48
Joda et al. (2019)	Journal Article	27	9	50
Ismail and Materwala (2020)	Journal Article	22	11	51 = 52
Shanbehzadeh et al. (2020)	Journal Article	22	11	52 = 51
Boyd et al. (2015)	Journal Article	36	5.14	53
van Olmen et al. (2020)	Journal Article	17	8.5	54
de Quiros et al. (2018)	Journal Article	23	5.75	55 = 56
Park et al. (2018)	Journal Article	23	5.75	56 = 55
Gamal et al. (2021)	Journal Article	11	11	57
de Moura Costa et al. (2020)	Journal Article	14	7	58
Genevieve et al. (2019)	Journal Article	16	5.33	59
Ammar et al. (2020)	Conference Proceedings	12	6	60 = 61
Samra et al. (2020)	Journal Article	12	6	61 = 60
Arul et al. (2024)	Journal Article	6	6	62
Ammar et al. (2021)	Journal Article	5	5	63

*Note*: For references Alkraiji et al. (2013), Tapuria et al. (2013), Ismail and Materwala (2020), Shanbehzadeh et al. (2020), de Quiros et al. (2018), Park et al. (2018), Ammar et al. (2020), Samra et al. (2020): the result of (a) × (b) results in a tie which means they share the same ranking.

### Publication analysis

According to Table 2, the 63 references selected for this study were represented across 45 different publications. Almost all of these publications were journals, with only two related to book series. The average number of references in these publications was 1.40. Regarding the citation analysis, *the Journal of the American Medical Informatics Association (JAMIA*) had the highest number of citations, as well as the highest average number of citations per year. *BMC Medical Informatics and Decision-Making* registered the highest average number of citations per article. *JMIR Formative Research* recorded the lowest score in the three previously reported metrics. A publication global impact ranking was also established, which resulted from the multiplication of four metrics: (1) average number of citations per year; (2) average number of citations per article; (3) 2021 CiteScore; (4) 2021 impact factor (Clarivate Analytics). The top five publications of this ranking were (1) *Journal of the American Medical Informatics Association (JAMIA*); (2) *Computational and Structural Biotechnology Journal (CSBJ)*; (3) *Nature Reviews Cardiology;* (4) *Health Affairs;* (5) *IEEE Journal of Biomedical and Health Informatics.* The lowest five positions of publication global impact raking were (1) *JMIR Formative Research;* (2) *Studies in Health Technology and Informatics;* (3) *Advances in Health Care Management;* (4) *Personal and Ubiquitous Computing;* (5) *Journal of Education and Health Promotion (JEHP).*

**Table 2.** Publication characteristics and global impact ranking.

Publication name	ISSN	Publication type	No. of references	Average citations per year (a)	Average citations per article (b)	2021 CiteScore (c)	Impact factor 2021 Journal Citation Reports (d)	Publication global impact ranking (a) × (b) × (c)^a^ × (d)^a^
Journal of the American Medical Informatics Association (JAMIA)	1527-974X	Journal	6	189.62	287	9.6	7.942	1
Computational and Structural Biotechnology Journal (CSBJ)	2001-0370	Journal	1	118.00	472	6	6.155	2
Nature Reviews Cardiology	1759-5010	Journal	1	20.50	41	31.9	49.421	3
Health Affairs	2694-233X	Journal	3	44.72	159	9.6	9.048	4
IEEE Journal of Biomedical and Health Informatics	2168-2208	Journal	1	24.11	217	10.9	7.021	5
Database (Oxford)	1758-0463	Journal	1	71.00	142	6.6	4.462	6
BMC Medical Informatics and Decision Making	1472-6947	Journal	1	34.43	482	4.6	3.298	7
Journal of Medical Systems	1573-689X	Journal	4	62.77	63	11.5	4.92	8
Globalisation and Health	1744-8603	Journal	1	20.80	104	8.7	10.401	9
Journal of Medical Internet Research	1438-8871	Journal	4	74.67	43	8.2	7.076	10
Journal of Biomedical Informatics (JBI)	1532-0480	Journal	2	31.23	86	8.2	8	11
npj Digital Medicine	2398-6352	Journal	1	19.00	39	11.8	15.357	12
JMIR mHealth and uHealth	2291-5222	Journal	1	33.00	99	8.2	4.947	13
BMC Health Services Research	1472-6963	Journal	3	48.17	164	3.9	2.908	14
Health Information Management Journal (HIMJ)	1833-3575	Journal	3	22.79	53	5.6	3.778	15
Health Systems & Reform (HS&R)	2328-8620	Journal	1	9.71	68	4.9	6.378	16
International Journal of Medical Informatics	1872-8243	Journal	1	5.53	83	8	4.73	17
Healthcare (Amsterdam, Netherlands)	2213-0772	Journal	1	19.75	79	2.5	3.16	18
BMC Medical Ethics	1472-6939	Journal	1	18.00	54	3.5	2.834	19
Sensors	1424-3210	Journal	1	8.40	42	6.4	3.847	20
Health Policy	1872-6054	Journal	1	6.22	56	5.2	3.255	21
BMJ Open	2044-6055	Journal	1	12.67	38	3.9	3.006	22
Applied Clinical Informatics Journal (ACI)	1869-0327	Journal	1	7.00	42	4	2.762	23
IEEE Access	2169-3536	Journal	1	5.75	23	6.7	3.476	24
Methods of Information in Medicine	2511-705X	Journal	1	12.00	24	4.5	1.8	25
Medical Archives	1986-5961	Journal	1	11.00	88	1.9	n.a.	26
International Journal of Population Data Science (IJPDS)	2399-4908	Journal	1	16.67	50	2.2	n.a.	27
PLOS ONE	1932-6203	Journal	1	5.33	16	5.6	3.752	28
Public Health Genomics	1662-8063	Journal	1	9.00	27	2.9	2.132	29
Healthcare Informatics Research (HIR)	2093-369X	Journal	1	5.44	49	5.1	n.a.	30
Online Journal of Issues in Nursing (OJIN)	1091-3734	Journal	1	13.43	94	n.a.	n.a.	31
Symmetry	2073-8994	Journal	1	11.00	22	4.3	n.a.	32
Perspectives in Health Information Management	1559-4122	Journal	1	8.75	105	1.1	n.a.	33
Acta Informatica Medica	1986-5988	Journal	1	9.00	36	2.7	n.a.	34
Wellcome Open Research	2398-502X	Journal	1	8.50	17	5.2	n.a.	35
Yearbook of Medical Informatics	2364-0502	Journal	1	5.75	23	4.8	n.a.	36
Journal of Healthcare Information Management (JHIM)	1943-734X	Journal	1	5.92	71	n.a.	n.a.	37
IEEE Transactions on Information Technology in Biomedicine	1558-0032	Journal	1	6.30	63	n.a.	n.a.	38
Health and Technology	2190-7196	Journal	1	7.00	14	3.2	n.a.	39
Medical Law International	2047-9441	Journal	1	6.11	55	0.9	n.a.	40
Journal of Education and Health Promotion (JEHP)	2319-6440	Journal	1	11.00	22	1.2	n.a.	41
Personal and Ubiquitous Computing	1617-4917	Journal	1	6.00	6	6.1	n.a.	42
Advances in Health Care Management: Health information technology in the international context	1474-8231	Book Series	1	6.00	60	0.6	n.a.	43
Studies in Health Technology and Informatics	1879-8365	Book Series	1	6.00	12	1.4	n.a.	44
JMIR Formative Research	2561-326X	Journal	1	5.00	5	1.8	n.a.	45

a This part of the formula is not applied if the metric is n.a.

### VOSviewer software title and abstract analysis

The database generated in Endnote was exported and evaluated using the VOSviewer software. The analysis was based on the information within the article’s title and abstract. Both abstract and title information are core information vessels within articles, crucial for article search optimisation within databases. Three dimensions were targeted for evaluation as they allowed an integrated analysis: (1) network visualisation, (2) overlay visualisation and (3) density visualisation. The first dimension showed the level of relationship between words, providing information about potential word clusters, and allowing their naming (via word analysis), for better understanding. The overlay visualisation showed which words and clusters were trending. The final dimension complemented the network visualisation by presenting simplified information about each word’s relevance.

The title network visualisation analysis found the existence of three clusters (Supplemental material, Figure S1, online supplement). The first cluster (*e-health information future trends*) consisted of the terms “blockchain technology,” “challenges,” “future direction” and “systematic review.” The second cluster (*e-health information uncertainty*) has in its composition the terms “challenge,” “development,” “health care” and “opportunity.” Terms such as “electronic health record,” “evaluation” and “healthcare” formed the final cluster (*e-health information quality assessment*).

When observing the same data display through the overlay visualisation (Supplemental material Figure S2, online supplement), the three clusters appear to have an increasing chronological timeline, as the *e-health information future trends* cluster appears to have been discussed later in 2020, while the *e-health information uncertainty* emerged mainly between 2015 and 2018. The oldest cluster was the *e-health information quality assessment*. The density analysis performed showed that the distribution of occurrences of words in all three clusters was similar. The *e-health information quality assessment* cluster was the most homogeneous, while the other two clusters were slightly heterogeneous (Supplemental material Figure S3, online supplement).

The abstract analysis (displayed in Figure S4, online supplement) revealed that increasing relationship between words when compared to the title analysis. In accordance with the title evaluation, the network visualisation analysis also presented three clusters. The first cluster (*e-health information stakeholder needs*) consists of the terms “EHR,” “electronic health record,” “hospital,” “integration,” “interoperability,” “lack,” “patient,” “standard” and “system”; the second cluster (*e-health information quality assessment*) has in its composition the terms “article,” “challenge,” “data,” “field,” “need,” “quality,” “research” and “researcher”; while the final cluster (*e-health information technological governance trends*) was related to “blockchain,” “privacy” and “security.”

In terms of timeline analysis (Supplemental material Figure S5, online supplement), the e*-health information technological governance trends* cluster was the most recent, while the other two clusters appeared to fall within the years 2015 and 2018. When the data were arranged by number of occurrences, the density analysis (Supplemental material Figure S6, online supplement) revealed that the most prominent words in the *e-health information stakeholder need* cluster were “interoperability” and “system,” with 48 and 44 occurrences, respectively. As for the *e-health information quality assessment* cluster, terms such as “data” (45 occurrences), “challenge” and “research” (both with 26 occurrences) formed the core of the cluster. “Privacy” (20 occurrences) was the leading word in the *e-health information technological governance trends* cluster.

**Figure 4.**
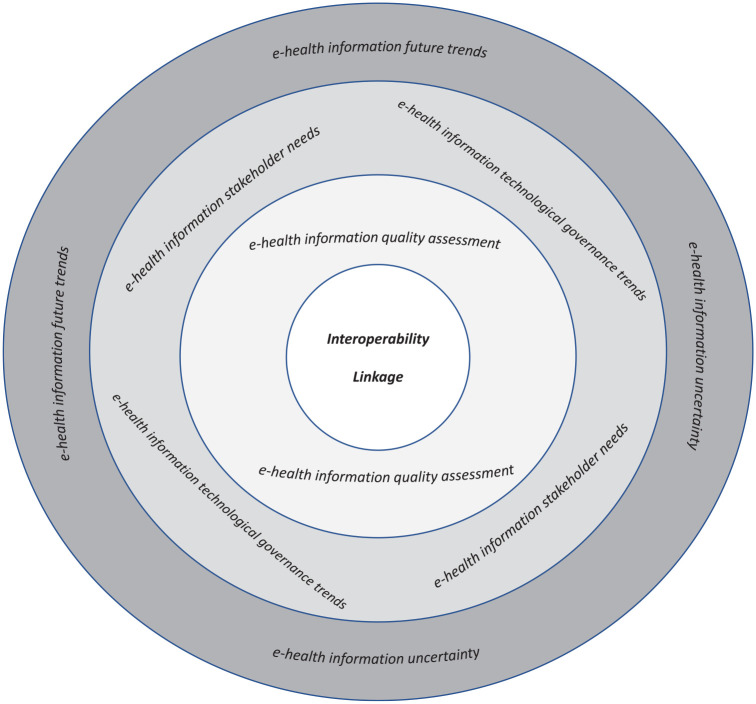
Theoretical framework to achieve high quality in interoperability and data linkage processes.

An integrated view of the cluster information retrieved through the VOSviewer software was also established (Figure 4). This original view of the title and abstract clusters demonstrated their interconnectivity pattern and relationship with system interoperability and data linkage.

### WORDSTAT software proximity of keywords analysis: interoperability versus linkage

**Figure 5.**
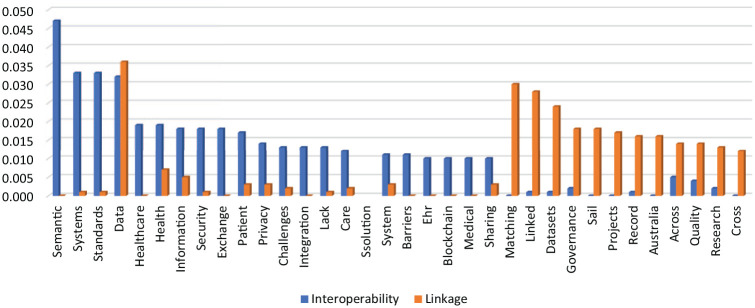
Proximity of keywords analysis by WORDSTAT: interoperability vs. linkage.

A proximity analysis of the selected two keywords by WORDSTAT software (Provalis Research, Montreal, Canada) was performed, and the data retrieved were analysed in Microsoft Excel (Figure 5). According to data retrieved, the terms most associated (association above 0.013) with “interoperability” were (1) semantic, (2) systems, (3) standards, (4) data, (5) healthcare, (6) health, (7) information, (8) security, (9) exchange, (10) patient, (11) privacy, (12) integration and (13) lack. As for the word “linkage,” the following results emerged: (1) data, (2) matching, (3) linked, (4) dataset, (5) governance, (6) sail, (7) projects, (8) record, (9) Australia, (10) across, (11) quality, (12) research. The only word with a similar proximity to the two words in the analysis was “data.”

## Discussion

### Sample timeline results

The sample’s timeline provided mixed results. While article numbers increased from years 2016 to 2020, they decreased abruptly in 2021. Conversely, global results containing abstracts continued to grow in 2021, suggesting that topics related to the scope of this research may have been redirected into other core subject areas, or that the COVID-19 pandemic may have resulted in quick solutions being incorporated into the marketplace with limited concern for data linkage and systems interoperability.

### Journal and article metrics

The reference global impact ranking metric attempts to eliminate the chronological bias that exists when only the number of citations of an article are taken into account. Usually, articles published earlier have an increased probability of achieving a higher number of citations, diminishing the importance of articles published more recently, with less time to accumulate possible citations. As the results of our study have demonstrated, the article citation metric does not correlate with the global impact ranking position; and that the ranking provides a more accurate method to evaluate the impact of research within the overall scope of this (data linkage and systems interoperability within health information management) research domain. The publication global impact ranking in our study produced similar results to the article global impact ranking, meaning the cross-combination of impact metrics and citation metrics did not correlate with standardised impact metrics. These findings suggest that worldwide acceptable metrics are not the best method to evaluate the influence and power of journals within specific disciplinary domains.

### Interoperability versus Linkage

In this study, interoperability and linkage concepts appeared distinct. The abstract analysis performed in the sample selection procedure showed that most abstracts contained the word “interoperability” while “linkage” was present in only a few. Only one abstract had both words. This simple analysis showed that researchers were giving more importance to interoperability than to linkage, and they either did not value or they overlooked the potential symbiotic power within the connection between these two disciplines. A further analysis that points to this same finding is the proximity of a keywords analysis performed in WORDSTAT software. The collected data showed that the term “interoperability” had a stronger association with a greater number of words when compared with the word “linkage.” The lack of association between these two words was also apparent, as only a few words achieved a strong association.

### VOSviewer cluster findings

The VOSviewer analysis originated three title clusters and three abstract clusters. As these clusters have interconnections, a theoretical framework was established as demonstrated in Figure 4. According to Figure 4, four levels were defined, representing different degrees of broadness. The outer layer is the most wide-ranging, and this complexity diminishes gradually until reaching the core. Interoperability and linkage issues in electronic health information management are uncertain as new trends emerge. To mitigate this uncertainty, governance models and stakeholders’ needs such as those explored by Witry et al. (2010), Alkraiji et al. (2013), Lavin et al. (2015), Abdekhoda et al. (2016) and Ammar et al. (2021) must be assessed so electronic health information possesses the highest degree of quality in which system interoperability and data linkage can achieve their purpose with efficacy and efficiency.

### Limitations and advantages

The current study had some limitations. First, in the data-gathering process, results exported from the three search platforms did not generate the same quality of information when imported into Endnote, meaning some crucial data were missing (e.g. some Google Scholar results were without year, abstract and publication information). Second, the criteria applied in the sample selection process, such as the one associated with article citations and selected keywords included in abstracts, may have enhanced the elimination of important articles. Third, sample data extracted from Endnote presented limitations when imported into software such as VOSviewer. However, this study has at least three advantages. To the best of our knowledge, this is the first bibliometric research in which electronic health information has intersected with both interoperability and linkage domains. Second, even with the above limitations, it was still possible to assess the quality and evolution of research about the subject of analysis. Finally, the current research provides insights into the main topics and concerns of the role of interoperability and linkage in health information systems.

## Conclusion

Results of this study have outlined theoretical and managerial implications of interoperability and linkage in health information management. One theoretical contribution is based on the need for more literature research about the combined role of interoperability and linkage in health information management, as the existent articles may suggest a lack of interest in the topic area. Also, new metrics and rankings were created to measure the real impact of articles and of journals within the scope of this research, minimising the biases provided by general impact factors and metrics that do not consider research subject specifications. In terms of managerial contributions, this research points to the necessity for the healthcare and information technology sectors to co-develop their solutions, to always consider linkage and interoperability concerns and to put the final consumer as a key player in their discussions. Moreover, these sectors should verify and evaluate stakeholders’ real-world needs so they can introduce their contributions to optimise the architecture of information technology solutions.

## Supplemental Material

sj-docx-1-him-10.1177_18333583241277952 – Supplemental material for System interoperability and data linkage in the era of health information management: A bibliometric analysis Supplemental material, sj-docx-1-him-10.1177_18333583241277952 for System interoperability and data linkage in the era of health information management: A bibliometric analysis by Tiago Costa, Teresa Borges-Tiago, Francisco Martins and Flávio Tiago in Health Information Management Journal

sj-docx-2-him-10.1177_18333583241277952 – Supplemental material for System interoperability and data linkage in the era of health information management: A bibliometric analysis Supplemental material, sj-docx-2-him-10.1177_18333583241277952 for System interoperability and data linkage in the era of health information management: A bibliometric analysis by Tiago Costa, Teresa Borges-Tiago, Francisco Martins and Flávio Tiago in Health Information Management Journal

sj-docx-3-him-10.1177_18333583241277952 – Supplemental material for System interoperability and data linkage in the era of health information management: A bibliometric analysis Supplemental material, sj-docx-3-him-10.1177_18333583241277952 for System interoperability and data linkage in the era of health information management: A bibliometric analysis by Tiago Costa, Teresa Borges-Tiago, Francisco Martins and Flávio Tiago in Health Information Management Journal

sj-docx-4-him-10.1177_18333583241277952 – Supplemental material for System interoperability and data linkage in the era of health information management: A bibliometric analysis Supplemental material, sj-docx-4-him-10.1177_18333583241277952 for System interoperability and data linkage in the era of health information management: A bibliometric analysis by Tiago Costa, Teresa Borges-Tiago, Francisco Martins and Flávio Tiago in Health Information Management Journal

sj-jpg-5-him-10.1177_18333583241277952 – Supplemental material for System interoperability and data linkage in the era of health information management: A bibliometric analysis Supplemental material, sj-jpg-5-him-10.1177_18333583241277952 for System interoperability and data linkage in the era of health information management: A bibliometric analysis by Tiago Costa, Teresa Borges-Tiago, Francisco Martins and Flávio Tiago in Health Information Management Journal

sj-jpg-6-him-10.1177_18333583241277952 – Supplemental material for System interoperability and data linkage in the era of health information management: A bibliometric analysis Supplemental material, sj-jpg-6-him-10.1177_18333583241277952 for System interoperability and data linkage in the era of health information management: A bibliometric analysis by Tiago Costa, Teresa Borges-Tiago, Francisco Martins and Flávio Tiago in Health Information Management Journal

sj-jpg-7-him-10.1177_18333583241277952 – Supplemental material for System interoperability and data linkage in the era of health information management: A bibliometric analysis Supplemental material, sj-jpg-7-him-10.1177_18333583241277952 for System interoperability and data linkage in the era of health information management: A bibliometric analysis by Tiago Costa, Teresa Borges-Tiago, Francisco Martins and Flávio Tiago in Health Information Management Journal

sj-jpg-8-him-10.1177_18333583241277952 – Supplemental material for System interoperability and data linkage in the era of health information management: A bibliometric analysis Supplemental material, sj-jpg-8-him-10.1177_18333583241277952 for System interoperability and data linkage in the era of health information management: A bibliometric analysis by Tiago Costa, Teresa Borges-Tiago, Francisco Martins and Flávio Tiago in Health Information Management Journal

sj-jpg-9-him-10.1177_18333583241277952 – Supplemental material for System interoperability and data linkage in the era of health information management: A bibliometric analysis Supplemental material, sj-jpg-9-him-10.1177_18333583241277952 for System interoperability and data linkage in the era of health information management: A bibliometric analysis by Tiago Costa, Teresa Borges-Tiago, Francisco Martins and Flávio Tiago in Health Information Management Journal

sj-jpg-10-him-10.1177_18333583241277952 – Supplemental material for System interoperability and data linkage in the era of health information management: A bibliometric analysis Supplemental material, sj-jpg-10-him-10.1177_18333583241277952 for System interoperability and data linkage in the era of health information management: A bibliometric analysis by Tiago Costa, Teresa Borges-Tiago, Francisco Martins and Flávio Tiago in Health Information Management Journal
