# Supplementary material for: System interoperability and data linkage in the era of health information management: A bibliometric analysis
Source: Health Inf Manag. 2024 Sep 16;54(3):214–26. doi: 10.1177/18333583241277952 (PMC12398637; doi:10.1177/18333583241277952)
Supplement: sj-docx-1-him-10.1177_18333583241277952 – Supplemental material for System interoperability and data linkage in the era of health information management: A bibliometric analysis [file sj-docx-1-him-10.1177_18333583241277952.docx]

**Supplementary materials**

**Tables (Supplemental)**

Table S1 - Search query results obtained in Google Scholar, PubMed, and Web of Science.

Table S2 - Type of publication analysis.

Table S3 - Sample overview analysis.

**Figures (Supplemental) (re-numbered)**

| Figure S1. | Title analysis performed by VOSviewer network visualisation |
| --- | --- |
| Figure S2. | Title analysis performed by VOSviewer overlay visualization |
| Figure S3. | Title analysis performed by VOSviewer density visualization |
| Figure S4. | Abstract analysis performed by VOSviewer network visualisation |
| Figure S5. | Abstract analysis performed by VOSviewer overlay visualization |
| Figure S6. | Abstract analysis performed by VOSviewer density visualization. |

----------------------------------------------------------------------------------------------------

**Note: 2 additional Figures added to list of supplemental (previously Figures 4 and 5, removed from the article and added to supplemental files. Previous supplemental files renamed so they are sequential in the order they are referred to in the text of the article**.

| **Previous Fig. #** | **New Fig #** | **Caption** |
| --- | --- | --- |
| **Figure 4** | **Figure S1.** | Title analysis performed by VOSviewer network visualisation |
| Figure S1 | **Figure S2.** | Title analysis performed by VOSviewer overlay visualization |
| Figure S2 | **Figure S3.** | Title analysis performed by VOSviewer density visualization |
| **Figure 5** | **Figure S4.** | Abstract analysis performed by VOSviewer network visualisation |
| Figure S3 | **Figure S5.** | Abstract analysis performed by VOSviewer overlay visualization |
| Figure S4 | **Figure S6.** | Abstract analysis performed by VOSviewer density visualization. |

Previous Figures (Supplemental files listing)

| ~~Figure S1.~~ | ~~Title analysis performed by VOSviewer network visualisation~~ |
| --- | --- |
| ~~Figure S2.~~ | ~~Title analysis performed by VOSviewer overlay visualization~~ |
| ~~Figure S3.~~ | ~~Title analysis performed by VOSviewer density visualization~~ |
| ~~Figure S4.~~ | ~~Abstract analysis performed by VOSviewer network visualisation~~ |
| ~~Figure S5.~~ | ~~Abstract analysis performed by VOSviewer overlay visualization~~ |
| Figure S6. | Abstract analysis performed by VOSviewer density visualization. |
