# Supplementary material for: System interoperability and data linkage in the era of health information management: A bibliometric analysis
Source: Health Inf Manag. 2024 Sep 16;54(3):214–26. doi: 10.1177/18333583241277952 (PMC12398637; doi:10.1177/18333583241277952)
Supplement: sj-docx-2-him-10.1177_18333583241277952 – Supplemental material for System interoperability and data linkage in the era of health information management: A bibliometric analysis [file sj-docx-2-him-10.1177_18333583241277952.docx]

| **Table S1.** Search query results obtained in Google Scholar, PubMed, and Web of Science. | | | | | | | | |
| --- | --- | --- | --- | --- | --- | --- | --- | --- |
| Google Scholar | | | PubMed | | | Web of Science | | |
| Search query | Search query results | Search query results exportation results | Search query | Search query results | Search query results exportation results | Search query | Search query results | Search query results exportation results |
| linkage OR interoperability "health data governance" | 294 | 280 | ("health data governance") AND (linkage OR interoperability) | 0 | 0 | (ALL=("health data governance")) AND ALL=(linkage OR interoperability) | 0 | 0 |
| linkage OR interoperability "health data management" | 1,340 | 972 | ("health data management") AND (linkage OR interoperability) | 93 | 93 | (ALL=("health data management")) AND ALL=(linkage OR interoperability) | 8 | 8 |
| linkage OR interoperability "health information governance" | 141 | 139 | ("health information governance") AND (linkage OR interoperability) | 0 | 0 | (ALL=("health information governance")) AND ALL=(linkage OR interoperability) | 0 | 0 |
| linkage OR interoperability "health information management" | 7,670 | 1,000 | ("health information management") AND (linkage OR interoperability) | 134 | 134 | (ALL=("health information management")) AND ALL=(linkage OR interoperability) | 51 | 51 |
| linkage OR interoperability "healthcare data governance" | 22 | 22 | ("healthcare data governance") AND (linkage OR interoperability) | 0 | 0 | (ALL=("healthcare data governance")) AND ALL=(linkage OR interoperability) | 0 | 0 |
| linkage OR interoperability "healthcare data management" | 561 | 540 | ("healthcare data management") AND (linkage OR interoperability) | 4 | 4 | (ALL=("healthcare data management")) AND ALL=(linkage OR interoperability) | 7 | 7 |
| linkage OR interoperability "healthcare information governance" | 9 | 9 | ("healthcare information governance") AND (linkage OR interoperability) | 0 | 0 | (ALL=("healthcare information governance")) AND ALL=(linkage OR interoperability) | 0 | 0 |
| linkage OR interoperability "healthcare information management" | 1,970 | 1,000 | ("healthcare information management") AND (linkage OR interoperability) | 3 | 3 | (ALL=("healthcare information management")) AND ALL=(linkage OR interoperability) | 4 | 4 |
| Total | 12,007 | 3,962 |  | 234 | 234 |  | 70 | 70 |
| EndNote results |  | 3,818 |  |  | 234 |  |  | 70 |
