# Supplementary material for: System interoperability and data linkage in the era of health information management: A bibliometric analysis
Source: Health Inf Manag. 2024 Sep 16;54(3):214–26. doi: 10.1177/18333583241277952 (PMC12398637; doi:10.1177/18333583241277952)
Supplement: sj-docx-3-him-10.1177_18333583241277952 – Supplemental material for System interoperability and data linkage in the era of health information management: A bibliometric analysis [file sj-docx-3-him-10.1177_18333583241277952.docx]

| **Table S2.** Type of publication analysis. | | | | | | | | |
| --- | --- | --- | --- | --- | --- | --- | --- | --- |
| Type of publication | Global results (without duplicates) | | With Abstract | | Abstract with "interoperability" and/or "linkage" | | Article number of citations per year index ≥ 4,94 (without exclusions) | |
| Book | 84 | 2.11% | - | - | - | - | - | - |
| Book Section | 284 | 7.14% | - | - | - | - | - | - |
| Conference Proceedings | 488 | 12.27% | 11 | 1.61% | 9 | 4.25% | 1 | 1.39% |
| Generic | 52 | 1.31% | - | - | - | - | - | - |
| Journal Article | 2884 | 72.52% | 674 | 98.39 | 203 | 95.75% | 71 | 98.61% |
| Report | 16 | 0.40% | - | - | - | - | - | - |
| Thesis | 169 | 4.25% | - | - | - | - | - | - |
| Total | 3799 | 100% | 685 | 100% | 212 | 100% | 72 | 100% |
| Note: Data extracted from EndNote and analysed in Microsoft Excel. | | | | | | | | |
