# Supplementary material for: System interoperability and data linkage in the era of health information management: A bibliometric analysis
Source: Health Inf Manag. 2024 Sep 16;54(3):214–26. doi: 10.1177/18333583241277952 (PMC12398637; doi:10.1177/18333583241277952)
Supplement: sj-docx-4-him-10.1177_18333583241277952 – Supplemental material for System interoperability and data linkage in the era of health information management: A bibliometric analysis [file sj-docx-4-him-10.1177_18333583241277952.docx]

| **Table S3.** Sample overview analysis. | | |
| --- | --- | --- |
| Reference | Abstract with "interoperability" and/or "linkage"? | In-house overview |
| Abdekhoda et al. (2016) | interoperability | Physician’s views and experiences about the use of electronic medical records |
| Ahmadi and Aslani (2018) | interoperability | A systematic review about the usefulness of cloud computing in implementing electronic health records |
| Ahmed et al. (2020) | interoperability | Literature review that discusses the role of artificial intelligence and machine learning in the field of medicine, including electronic health records |
| Alkraiji et al. (2013) | interoperability | Healthcare decision-makers perspectives on the barriers to the adoption of health data standards |
| Alonso et al. (2019) | interoperability | Literature review that discusses blockchain use in eHealth, namely electronic health and medical reports |
| Ammar et al. (2020) | interoperability | The role of personal health libraries as patients’ private database to assist in their health issues decision making process |
| Ammar et al. (2021) | interoperability | Development of personal health libraries and mobile applications according to the requirements of users |
| Arul et al. (2021) | interoperability | The role of blockchain applied to the internet of medical things with a proposal of a multi-modal secure data dissemination framework. |
| Avila et al. (2017) | interoperability | Applicability of service-oriented architecture to health systems as a tool for systems integration efficiency |
| Bahga and Madisetti (2013) | interoperability | Design of a cloud-based interoperable electronic health record system (CHISTAR) |
| Bates (2005) | interoperability | Barriers and policies influencing the adoption of electronic health records by U.S. physicians |
| Blasimme et al. (2018) | Interoperability | Review of policy documents about data-sharing guidance |
| Blazona and Koncar (2007) | Interoperability | Application of the health level 7 (HL7) standard to a radiology information system for data integration into hospital information networks. |
| Boyd et al. (2015) | Linkage | Linkage of different health data records into a national data linkage infrastructure in Australia |
| de Moura Costa et al. (2020) | Interoperability | Systematic literature review of fog computing application to healthcare |
| de Quiros et al. (2018) | Interoperability | Development of a hospital’s interoperable health information system through a terminology server and its standard terminologies |
| Detmer et al. (2008) | Interoperability | Determinants of implementing an integrated personal health record framework |
| Dinh-Le et al. (2019) | Interoperability | Overview and review of the integration process between electronic health records and wearable health technology |
| Downs et al. (2019) | Linkage | Data linkage project between clinical mental health service data and education and social care services data |
| Dubovitskaya et al. (2020) | Interoperability | Development of blockchain-based electronic health records data sharing and management, which involves actively the patient and caregivers |
| Durneva et al. (2020) | Interoperability | Systematic review of the application of blockchain technology in patient care |
| Edwards et al. (2010) | Interoperability | Literature review about barriers to health information exchange |
| Ford et al. (2009) | Linkage | Establishment of a research databank (SAIL) of health and well-being personal data that safely gathers information from different types of data sources |
| Furukawa et al. (2013) | Interoperability | Internal and external exchange trends of hospital electronic health data |
| Gamal et al. (2021) | Interoperability | Overview and review of the current state of database management systems that stores standardized electronic health record data |
| Genevieve et al. (2019) | Interoperability & Linkage | Systematic review of the barriers and enablers of health data sharing, linkage, and harmonization |
| Gordon and Catalini (2018) | Interoperability | The role of blockchain technology in patient-centred interoperability |
| Huang et al. (2017) | Interoperability | Study cases about the implementation of information systems in the governmental healthcare sector and its challenges |
| Hussien et al. (2019) | Interoperability | A systematic review about blockchain technology in the Healthcare sector |
| Hylock and Zeng (2019) | Interoperability | Application of blockchain technology to a patient-centric framework as a promoter of consistent and comprehensive medical records |
| Ismail and Materwala (2020) | Interoperability | Evaluation of the performance of a blockchain-based and patient-centric healthcare data management platform versus the traditional hospital data management |
| Joda et al. (2019) | Interoperability | Health data collection, sharing, analytics, and artificial intelligence in oral healthcare practice and dental research |
| Johnson et al. (2008) | Linkage | A provider-centric electronic health records platform proposal that incorporates narrative data and coded data |
| Jones et al. (2014) | Linkage | Overview of the SAIL gateway (remote access) for access to a national architecture for e-health research and evaluation |
| Jones et al. (2019) | Linkage | Operational overview of the SAIL databank as a health and well-being data source for research |
| Kalkman et al. (2019) | Linkage | Systematic review of the principles and norms (governance) of responsible data sharing in health research |
| Kaplan and Harris-Salamone (2009) | Interoperability | Overview and discussion of the determinants of implementation success or failure of health information technology in healthcare systems |
| Kasthurirathne et al. (2015) | Interoperability | Improvement of an electronic medical record system’s interoperability using the fast healthcare interoperability resources (FHIR) standard instead of the Health Level 7 (HL7) standard. |
| Kharrazi et al. (2017) | Interoperability | Overview and definition of population health informatics, including its data governance and management, interoperability, and standards |
| Koppel and Lehmann (2015) | Interoperability | Advantages and disadvantages of having a unique electronic health record platform in hospitals and health systems. |
| Krittanawong et al. (2020) | Interoperability | Integrated application of blockchain and artificial intelligence to health data in the field of cardiovascular medicine |
| Lavin et al. (2015) | Interoperability | Nurses’ views and experiences about the use of electronic health records |
| Lee et al. (2020) | Interoperability | Development of an international personal health records management platform which is based on blockchain technology |
| Lucyk et al. (2017) | Linkage | Barriers and challenges to obtain administrative health data of high-quality |
| Mandel et al. (2016) | Interoperability | Development of an electronic health record platform based on FHIR standard |
| Masud et al. (2012) | Interoperability | Data interoperability among heterogenic patient-centric data and its sources |
| Mohammadzadeh and Safdari (2014) | Interoperability | Opportunities and challenges of mobile health technology in chronic disease management |
| Oderkirk et al. (2013) | Linkage | Use and linkage of personal health data and its challenges in different countries. |
| Park et al. (2018) | Interoperability | Creation of a smart emergency medical service based on the needs of paramedics through the development of an interoperable system that connects electronic medical records and data from monitoring devices |
| Rezaeibagha et al. (2015) | Interoperability | Literature review that discusses the requirements for electronic health records in terms of security, privacy, and standards |
| Salas-Vega et al. (2015) | Interoperability | European policymakers pose challenges in the application of big data in healthcare |
| Samra et al. (2020) | Interoperability | Overview of hospital databases' contribution to medical research by interviewing mostly healthcare professional |
| Sethi and Laurie (2013) | Linkage | Proportional information governance in health data linkage for research purposes |
| Shanbehzadeh et al. (2020) | Interoperability | Interoperable reporting framework for COVID-19, which includes levels of administrative data and clinical data |
| Sinaci et al. (2020) | Interoperability | Development of FAIR (findability, accessibility, interoperability, and reusability) principles-based framework using Health Level 7 FHIR standard for health data research |
| Sittig et al. (2018) | Interoperability | Shared stakeholders’ responsibility for the safety of electronic health records |
| Tapuria et al. (2013) | Interoperability | Role of clinical archetypes in achieving semantic interoperability of electronic health records |
| van Olmen et al. (2020) | Linkage | Barriers in the implementation of mobile health platforms |
| Vazirani et al. (2020) | Interoperability | Role of blockchain in the management of electronic health records |
| Vest (2012) | Interoperability | Overview of health information exchange application in an international context |
| Warner et al. (2016) | Interoperability | Development of a mobile application based on the HL7 FHIR standard, which provides genomic health information to oncologists and their patients in real-time for precision medicine definition |
| Witry et al. (2010) | Interoperability | Physician’s views about the possible benefits and concerns in the use of personal health records from providers and patients |
| Wollersheim et al. (2009) | Interoperability | Literature review about archetype-based electronic health records relevant to health information managers |
| Note: All references are journal articles except for Ammar et al. (2020) (which is a conference proceeding). | | |
