# Supplementary figures and images for: System interoperability and data linkage in the era of health information management: A bibliometric analysis
Source: Health Inf Manag. 2024 Sep 16;54(3):214–26. doi: 10.1177/18333583241277952 (PMC12398637; doi:10.1177/18333583241277952)

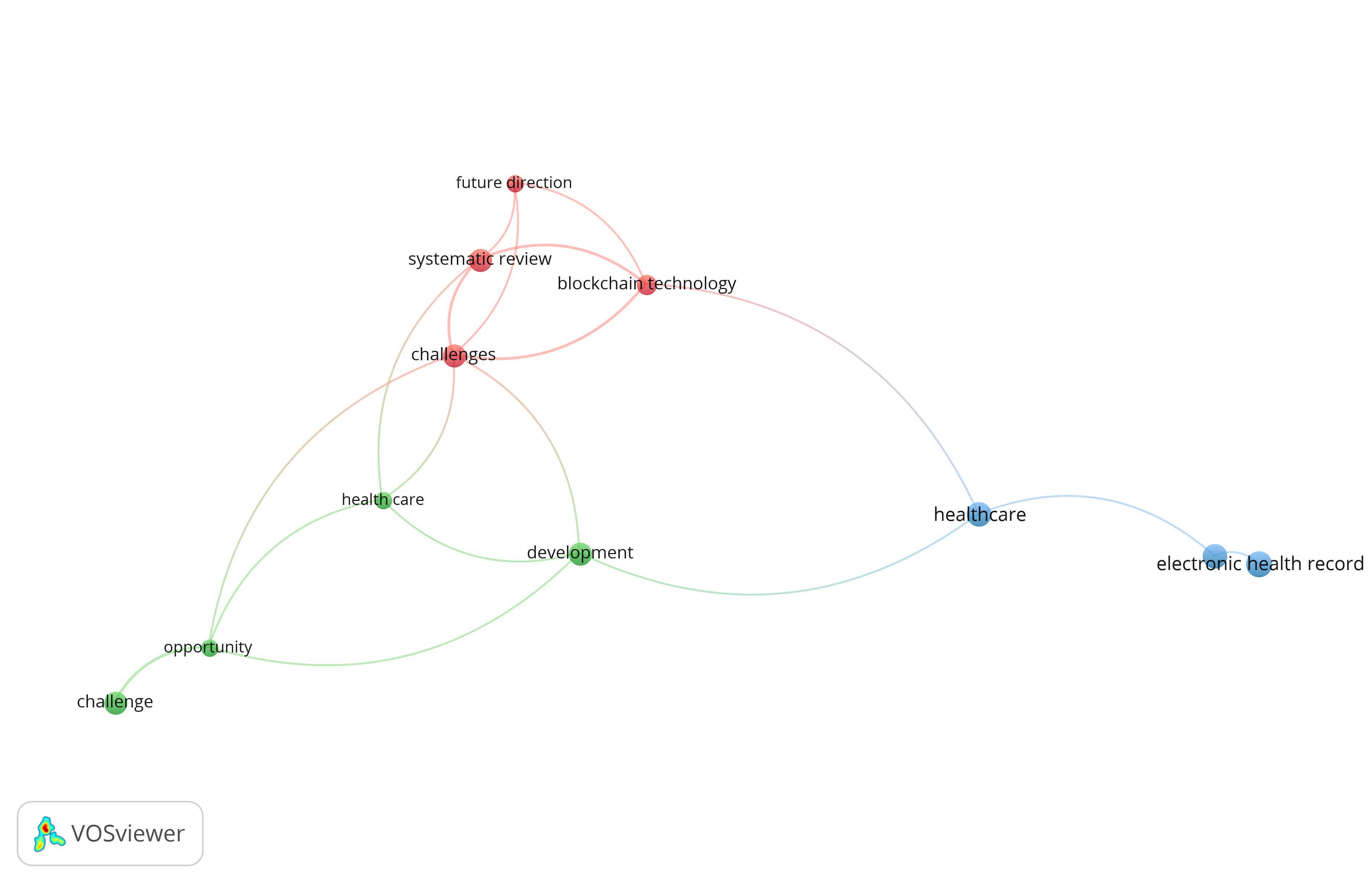

Supplement: sj-jpg-5-him-10.1177_18333583241277952 – Supplemental material for System interoperability and data linkage in the era of health information management: A bibliometric analysis [file sj-jpg-5-him-10.1177_18333583241277952.jpg]

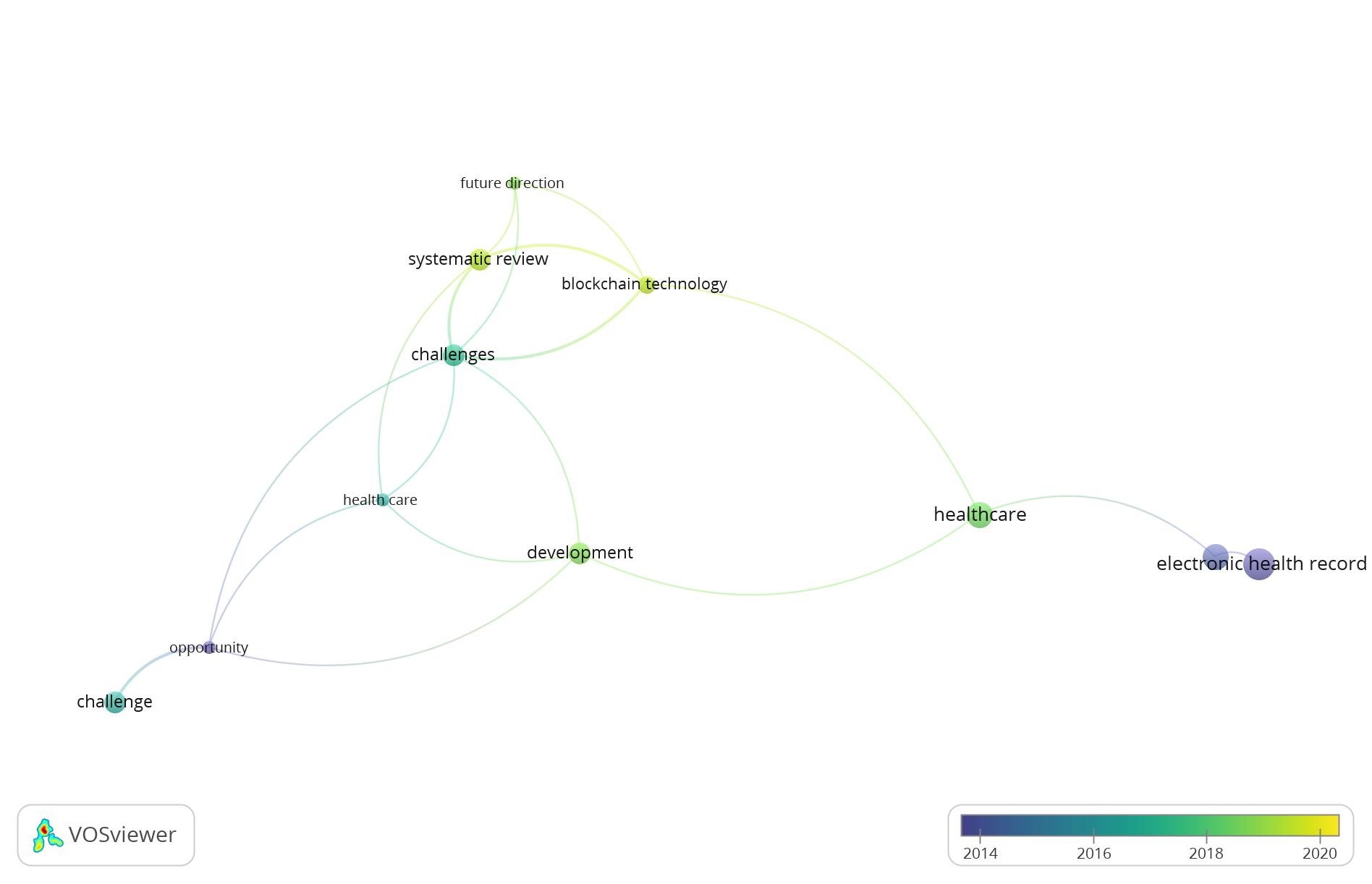

Supplement: sj-jpg-6-him-10.1177_18333583241277952 – Supplemental material for System interoperability and data linkage in the era of health information management: A bibliometric analysis [file sj-jpg-6-him-10.1177_18333583241277952.jpg]

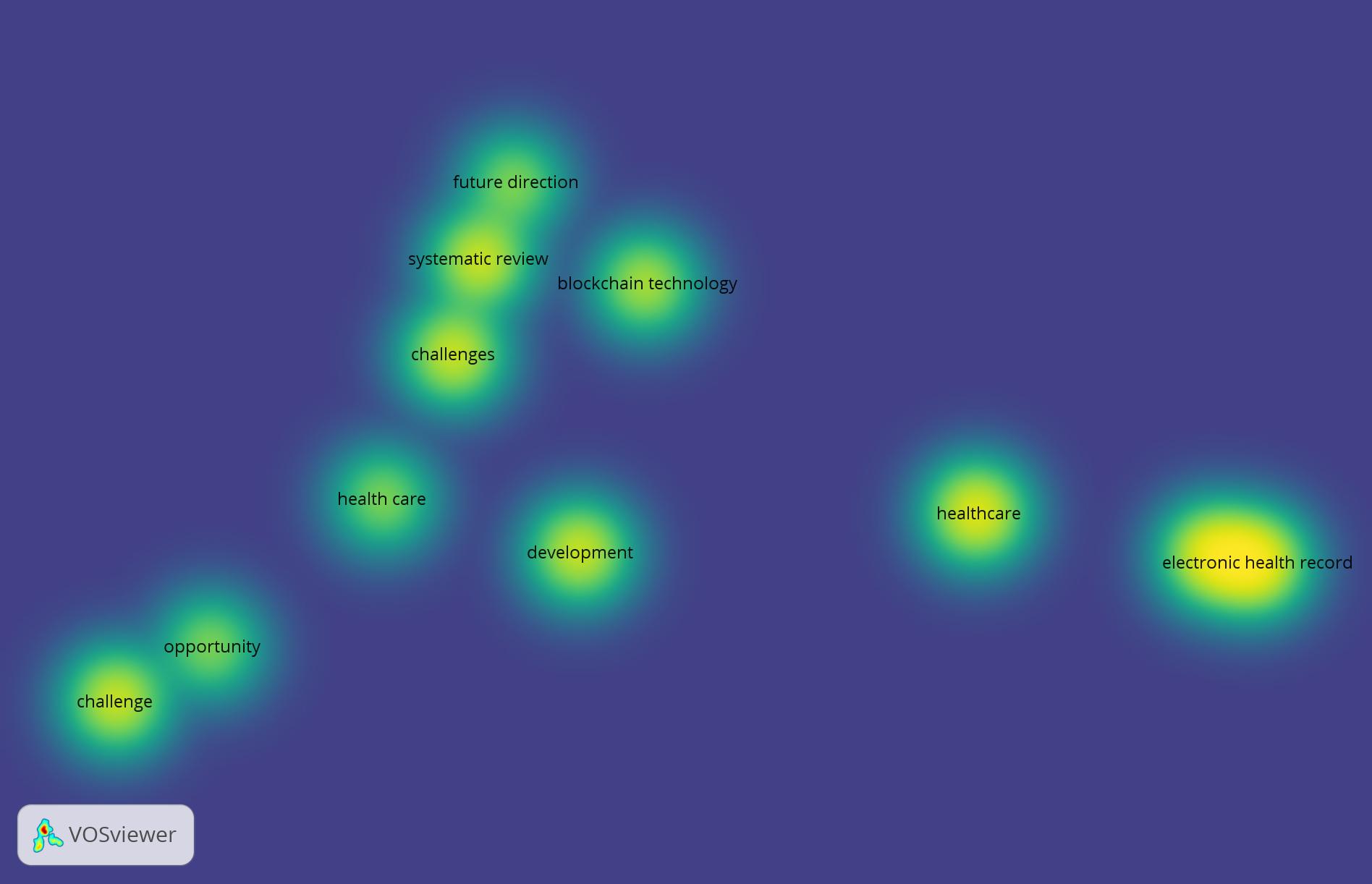

Supplement: sj-jpg-7-him-10.1177_18333583241277952 – Supplemental material for System interoperability and data linkage in the era of health information management: A bibliometric analysis [file sj-jpg-7-him-10.1177_18333583241277952.jpg]

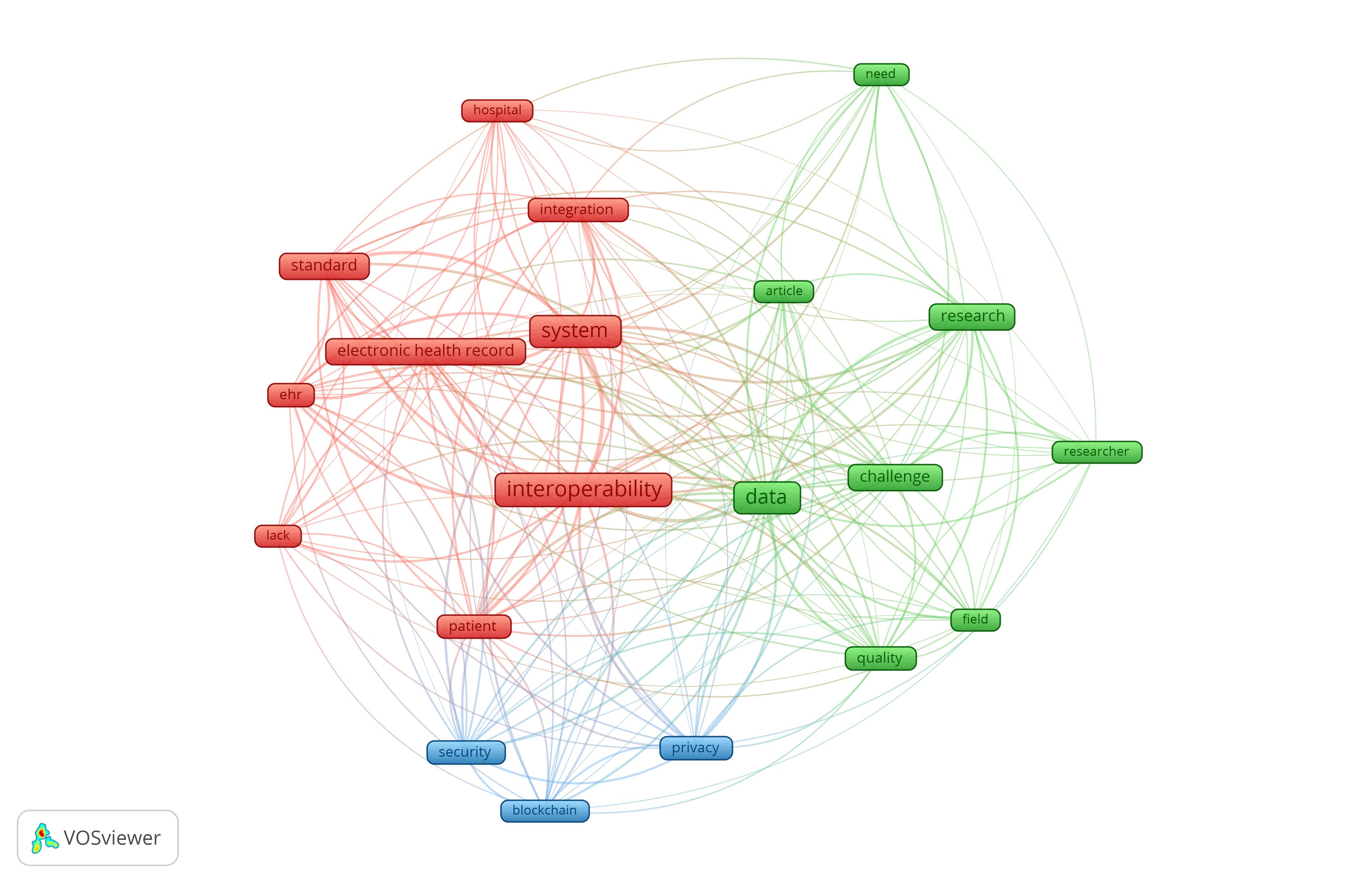

Supplement: sj-jpg-8-him-10.1177_18333583241277952 – Supplemental material for System interoperability and data linkage in the era of health information management: A bibliometric analysis [file sj-jpg-8-him-10.1177_18333583241277952.jpg]

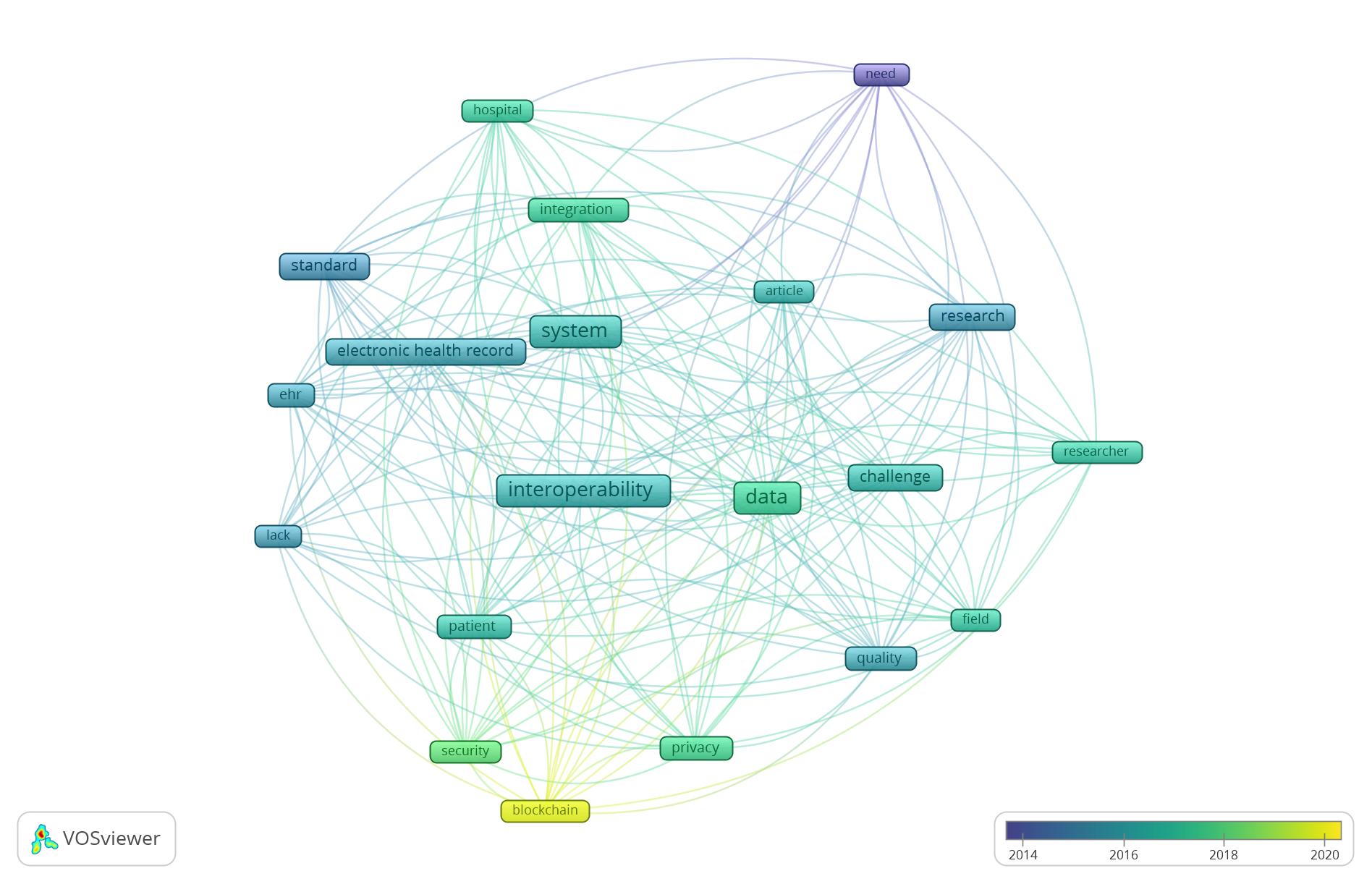

Supplement: sj-jpg-9-him-10.1177_18333583241277952 – Supplemental material for System interoperability and data linkage in the era of health information management: A bibliometric analysis [file sj-jpg-9-him-10.1177_18333583241277952.jpg]

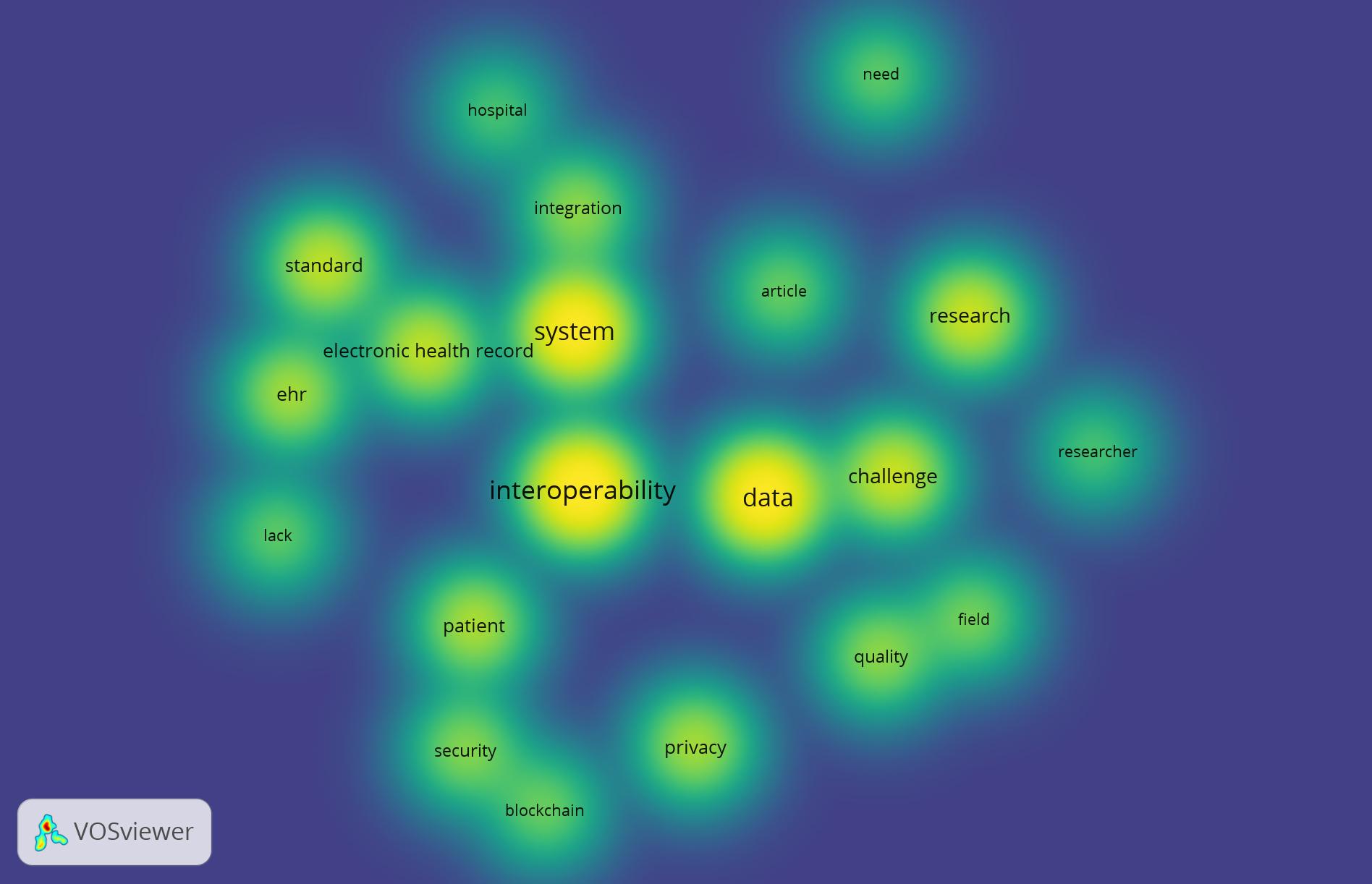

Supplement: sj-jpg-10-him-10.1177_18333583241277952 – Supplemental material for System interoperability and data linkage in the era of health information management: A bibliometric analysis [file sj-jpg-10-him-10.1177_18333583241277952.jpg]
